# Early-Middle Pleistocene benthic turnover and oxygen isotope stratigraphy from the Central Mediterranean (Valle di Manche, Crotone Basin, Italy): Data and trends

**DOI:** 10.1016/j.dib.2018.02.017

**Published:** 2018-02-15

**Authors:** Michele Azzarone, Patrizia Ferretti, Veronica Rossi, Daniele Scarponi, Luca Capraro, Patrizia Macrì, John W. Huntley, Costanza Faranda

**Affiliations:** a Dipartimento di Scienze Biologiche, Geologiche e Ambientali, Università di Bologna, Piazza di Porta San Donato 1, I-40126 Bologna, Italy; b Consiglio Nazionale delle Ricerche, Istituto per la Dinamica dei Processi Ambientali (CNR-IDPA), Via Torino 155, I-30172, Venezia Mestre, Italy; c Dipartimento di Geoscienze, Università di Padova, Via G. Gradenigo 6, I-35131 Padova, Italy; d Istituto Nazionale di Geofisica e Vulcanologia, Via di Vigna Murata 605, I-00143 Roma, Italy; e Department of Geological Sciences, University of Missouri, 101 Geology Building, Columbia, MO 65211, USA; f Dipartimento di Scienze, University of Roma Tre, Largo San Leonardo Murialdo 1, 00146 Roma, Italy

## Abstract

Ostracod faunal turnover and oxygen isotope data (foraminifera) along the Valle di Manche (VdM) section are herein compiled. Specifically, the material reported in this work includes quantitative palaeoecological data and patterns of ostracod fauna framed within a high-resolution oxygen isotope stratigraphy (δ^18^O) from *Uvigerina peregrina*. In addition, the multivariate ostracod faunal stratigraphic trend (nMDS axis-1 sample score) is calibrated using bathymetric distributions of extant molluscs sampled from the same stratigraphic intervals along the VdM section. Data and analyses support the research article “Dynamics of benthic marine communities across the Early-Middle Pleistocene boundary in the Mediterranean region (Valle di Manche, Southern Italy): biotic and stratigraphic implications” Rossi et al. [1].

**Specifications Table**

**Table:** 

Subject area	*Earth Science*
More specific subject area	*Palaeoecology and Oxygen Isotope Stratigraphy*
Type of data	*Tables, Figures and Text file*
How data were acquired	*Field and dissecting microscope observations. Isotope ratio mass spectrometry*
Data format	*Raw and analysed*
Experimental factors	
Experimental features	
Data source location	*San Mauro Marchesato (Crotone, Southern Italy)*
Data accessibility	*The data are available with this article*

**Value of the data**

• Valle di Manche (VdM) is a key-section within the Mediterranean Basin as it straddles the Early-Middle Pleistocene boundary and contains a record of the Matuyama–Brunhes reversal. The abundance data of benthic organisms here presented complement the available documentation for the VdM section.
• The multidisciplinary approach adopted provides a viable strategy for quantifying stratigraphic and palaeontological patterns, which allowed for an improved reconstruction of depositional environments.
• The data here presented could be compared to other Mediterranean siliciclastic successions that record Early-Middle Pleistocene high frequency sea level fluctuations.

## 1 Data

We report data from ostracod fauna (39 samples, >3600 valves; Appendix 1) and stable isotope data from the benthic foraminifera *Uvigerina peregrina* sampled at high resolution along the 38m-thick investigated interval of the Valle di Manche section (Crotone Basin, Southern Italy [2], [3]).

## 2 Experimental design, materials and methods

Concerning the ostracod fauna, each valve was counted as one individual (Appendix 1). *Uvigerina peregrina* specimens were picked from the >150 μm coarse fraction of 229 sediment samples (Table 2 in [3]), which were previously disaggregated using distilled water.

### 2.1 Unconstrained gradient analysis

**Fig. 1:**
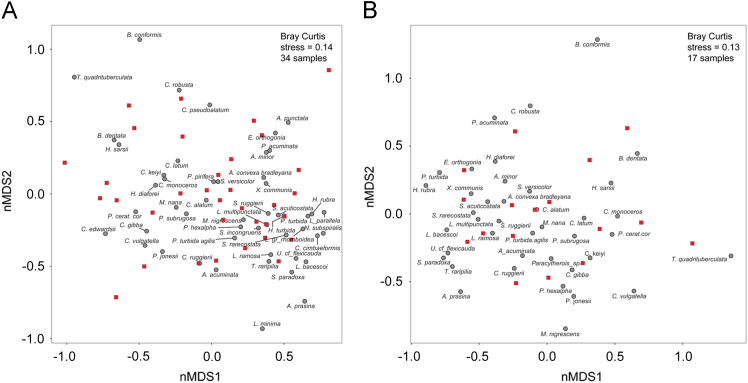
Non-metric multidimensional scaling outputs performed on data matrices with different taxonomic and numerical resolution. A) Samples ≥20 specimens and species recorded in more than one sample (i.e., 34 samples/51 species matrix). B) Samples ≥20 specimens and species recorded in more than two samples (i.e., 17 samples/34 species matrix; Fig. 1B). Square and circle symbols represent sample and species, respectively.

**Fig. 2:**
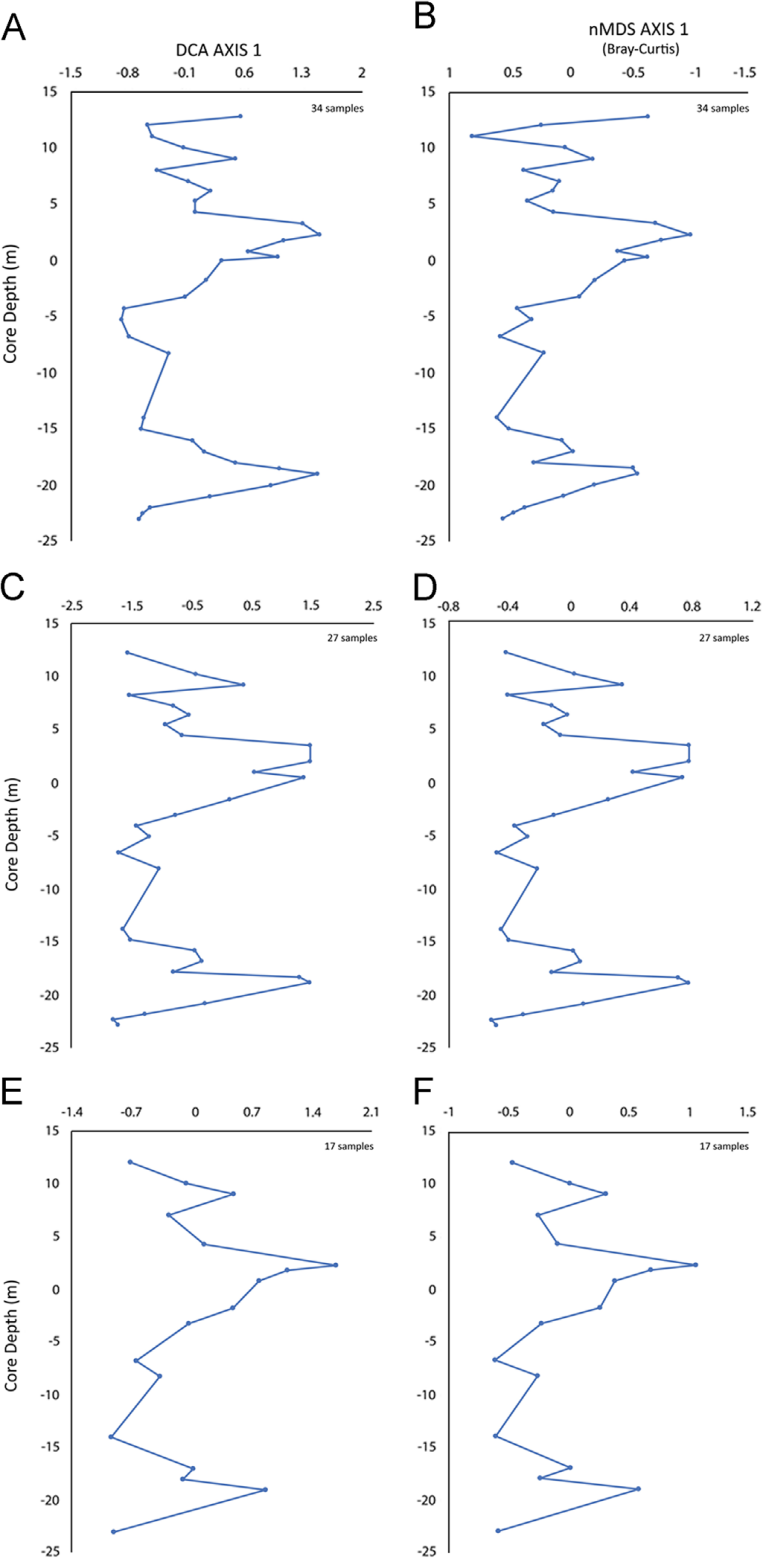
Multiple stratigraphic plots of Detrended Correspondence Analysis (A, C, E) and non-Metric Multidimensional Scaling (B, D, F) axis 1 sample scores. A-B) Sample ≥20 specimens and species singletons excluded. C-D) Sample ≥25 specimens and species occurrence ≥5 samples. E-F) Ostracod dataset comparable (in sample size and sampling resolution) to the mollusc dataset reported in [13]; sample size ≥20 specimens and species singletons excluded.

Detrended correspondence analysis (DCA) and non-metric multi-dimensional scaling (nMDS) are two widely employed indirect ordination methods in palaeoecology. As both ordination techniques have different strengths and weaknesses, the best approach is to use both methods as a crosscheck on the robustness of the outputs [4], [5]. Faunal counts were log-transformed to prevent distortion due to very abundant species. Then, DCA and nMDS were performed on a set of abundance matrices derived varying sample and taxon thresholds. In this work, we focus on nMDS outputs (2-dimensions and based on Bray-Curtis distance; Fig. 1, Table 1A), as for DCA outputs we refer to [1]. Stratigraphic plots of nMDS and DCA axis-1 sample scores are also displayed (Table 1A; Fig. 2 A, C, E and B, D, F respectively). Ordination analyses were performed in R 3.3.2 [6] with “vegan” package and PAST software [7].

**Table 1:** A - Sample information and major axis sample scores obtained from non-Metric Multidimensional Scaling (nMDS) and Detrended Correspondence Analysis (DCA) on Valle di Manche ostracod and mollusc datasets. A1) Mollusc sample label. A2) DCA axis 1 sample score; A3) Stratigraphic offset with respect to the adjacent ostracod sample. A4) Ostracod sample label. A5–6) nMDS axis 1 sample score obtained from a reduced ostracod matrix (employing absolute—Abs and relative—Rel abundances) comparable to the mollusc one (i.e., 17 samples see Scarponi et al., 2014). Stress values = 0.19 and 0.16, respectively. A7–8) As for A5–6 but employing DCA. A9–10) nMDS axis 1 sample score obtained from the 51×34 ostracod matrix employing absolute—Abs and relative—Rel abundances. Stress values = 0.20 and 0.19, respectively. A11–12) As for A9–10 but employing DCA. B. Linear correlation (RMA) coefficients (r—Pearson) and p-values (α=0.05) between ordination of ostracod matrices (i.e., DCA- 1 or nMDS-1) and mollusc DCA axis 1 sample score (*after*[8]).

**A) Ordination analyses and sample information from the Valle di Manche section**														
			**Ostracod samples**											
*after*Scarponi et al. (2014)					**Matrix 17 samples**						**Matrix 34 samples**			
**Label**	**DCA-1**	**S-offset**	**Label**		**nMDS-1**				**DCA-1**		**nMDS-1**		**DCA-1**	
		**(cm)**			**Abs**		**Rel**		**Abs**	**Rel**	**Abs**	**Rel**	**Abs**	**Rel**
**1)**	**2)**	**3)**	**4)**		**5)**		**6)**		**7)**	**8)**	**9)**	**10)**	**11)**	**12)**
Bk22	196	20	SMA50		-0.24433		-0.25854		22	0	0.121	0.128	15	31
Bk21	117	0	SMA42		-0.05252		-0.04989		87	73	0.012	0.014	94	74
Bk20	95	-40	SMA38		0.14789		0.084843		143	127	-0.067	-0.047	135	137
Bk19	122	0	SMA30		-0.10886		-0.10742		67	57	0.051	0.052	79	80
Bk18	67	-10	SMA18		-0.03471		-0.02284		109	61	0.043	0.042	83	88
Bk17	0	40	SMA10		0.41237		0.41683		264	237	-0.307	-0.282	255	238
Bk16	9	-30	SMA8		0.31321		0.33418		206	218	-0.245	-0.237	235	195
Bk15	51	0	SMA4		0.16096		0.18789		173	151	-0.133	-0.160	174	152
Bk14	90	40	SMA-8		0.073954		0.078073		142	106	-0.051	-0.072	118	102
Bk13	98	-20	SMA-14		-0.16361		-0.16797		91	41	0.055	0.048	80	76
Bk12	223	10	SMB14		-0.18192		-0.21069		29	11	0.145	0.145	20	8
Bk11	198	10	SMB20		-0.14185		-0.15454		56	14	0.090	0.095	38	56
Bk9	164	30	SMB40		-0.24981		-0.20625		0	30	0.117	0.096	45	26
Bk8	80	20	SMB52		-0.04612		-0.05474		96	70	0.026	0.019	91	99
Bk7	59	10	SMB56		-0.02318		0.030985		84	157	0.037	0.025	134	137
Bk6	4	0	SMB60		0.35609		0.32507		181	257	-0.224	-0.229	267	236
Bk5	272	60	SMB76		-0.21757		-0.22499		2	5	0.136	0.136	24	20

**B)** Linear correlation: ordination axis 1 ostracod-sample scores *vs*. DCA axis 1 mollusc-sample score														
Ostracod (17 samples matrix) *vs.* Mollusc matrix								Ostracod (34 samples matrix) *vs.* Mollusc matrix						
*nMDS-1 absolute abundance*				r = -0.844, p«0.05				*nMDS-1 absolute abundance*					r = 0.849, p«0.05	
*nMDS-1 relative abundance*				r = -0.873, p«0.05				*nMDS-1 relative abundance*					r = 0.864, p«0.05	
*DCA-1 log-transformed raw values*				r = -0.881, p«0.05				*DCA-1 log-transformed raw value*					r = 0.894, p«0.05	
*DCA-1 relative abundance*				r = -0.880, p«0.05				*DCA-1 relative abundance*					r = -0.905, p«0.05	

### 2.2 Ostracod and mollusc faunal trends along Valle di Manche (VdM) section

Reduced Major Axis (RMA) regression was performed to explore the relationship between ostracod and mollusc faunal composition along the Valle di Manche section (Table 1). The multiple DCA and nMDS axis 1 sample scores obtained from ostracods (Table 1A) were correlated via RMA to the scores previously obtained from DCA on the mollusc matrix (see [8]; Table 1A). All analyses returned high and significant correlation coefficients (Table 1B).

### 2.3 Oxygen isotope stratigraphy and age model

Between 10 and 15 specimens of *U. peregrina* were analysed in order to reduce statistical variability. After being lightly crushed, to remove organic contaminants, the selected specimens were soaked in hydrogen peroxide (3%). Then, analytical grade acetone was added, and the samples cleaned ultrasonically, after which the excess liquid was removed. All stable isotope analyses were carried out on an automated continuous flow carbonate preparation GasBench II device, attached to a Thermo Scientific Delta V Advantage Isotope Ratio Mass Spectrometer. Measurements of δ^18^O were determined relative to the Vienna Peedee belemnite (VPDB) standard, with an analytical precision that is better than 0.1‰.

The chronology for the Valle di Manche section was developed by tuning the *Uvigerina peregrina* δ^18^O signal to the stacked planktonic oxygen isotope record derived from the Mediterranean Sea [9], [10]. In the initial stages, we produced an alternative age model by making use of the time scale of Konijnendijk and collaborators [11], which is also based on a stacked and averaged suite of oxygen isotope records from the eastern Mediterranean, in this case from benthic foraminifera. This initial tuning approach was based on the assumption that the correlation of the benthic δ^18^O signal from the VdM succession to a benthic record from the Mediterranean region appeared to be a more advisable choice than the use of a planktonic δ^18^O stack as a tuning target. However, the benthic δ^18^O from VdM and the benthic δ^18^O stack of [11] have little in common at either low or high frequency, as the suite of cores used by [11] reflects the dynamics of different (i.e. deeper) water masses. Serious discrepancies between the dataset from VdM and the benthic δ^18^O stack in the time interval from ca. 860 to 815 ka (MIS 21), lead to difficulties in developing a tuned timescale (see Figure 10 in [3]). This is an interval when some sources of uncertainty arise in the time scale developed by [11], as changes in insolation forcing are generally relatively small between 700–950 ka, no sapropel layers are present, and proxies lack a characteristic pattern to tie to insolation, making the resulting chronology dubious [12]. For these reasons, this initial age model was rejected.

**Fig. 3:**
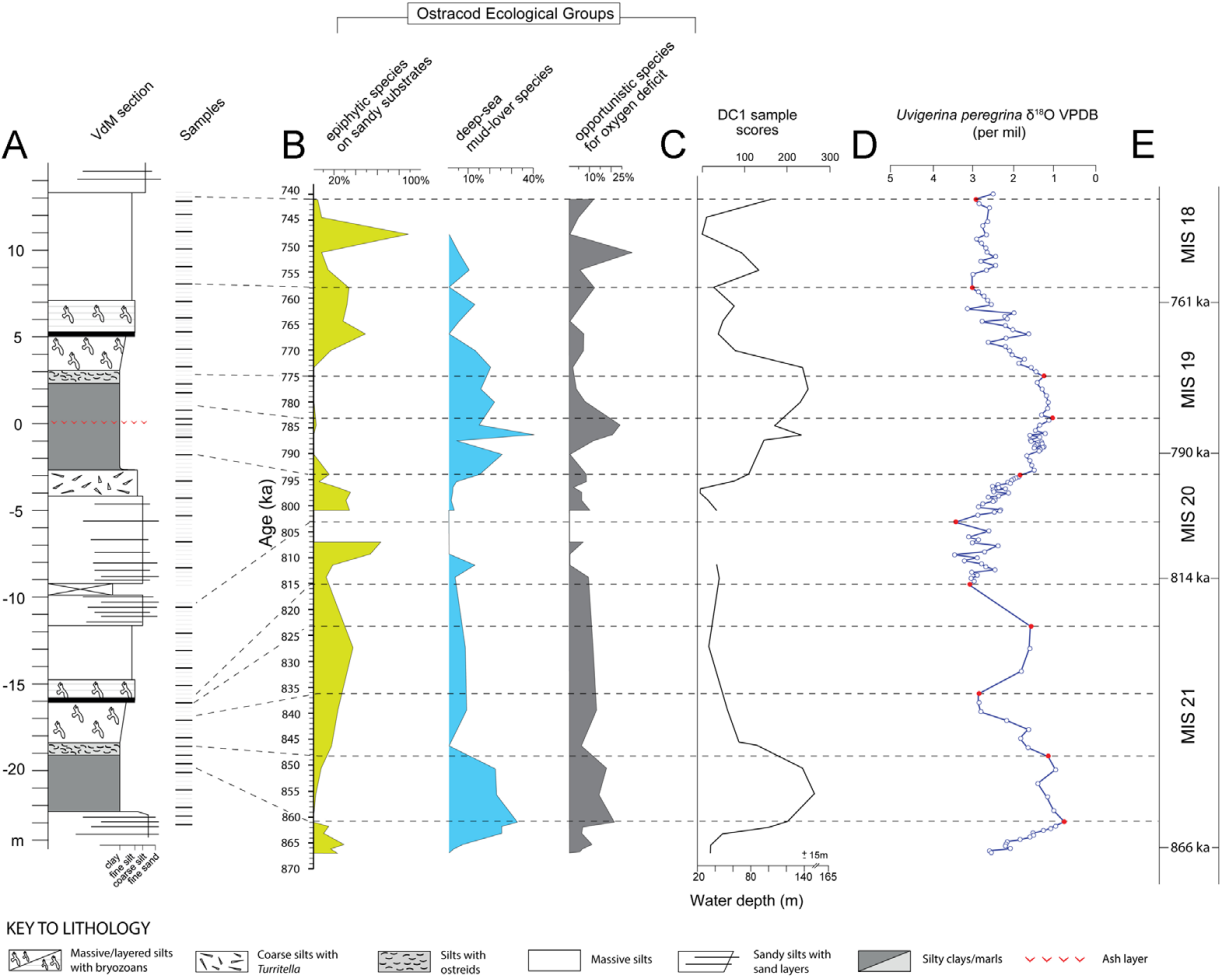
Data summary of the high-resolution chronostratigraphic and palaeoenvironmental inferences retrieved at Valle di Manche (VdM). A) Physical stratigraphy of VdM section along with location of the 229 collected samples, in bold the 39 samples analysed for the ostracod fauna. B) Ostracod ecological groups distinguished on the basis of different ecological preferences, in terms of substrate and oxygen conditions, of the species recorded along the VdM section. C) Stratigraphic pattern in DCA-calibrated water depth based on the 34×51 ostracod matrix (see also Fig. 2A). D) *U. peregrina* oxygen isotope stratigraphy of the VdM section. E) Marine Isotope Stages (MIS) straddling the Early-Middle Pleistocene transition. Red dots represent the control points employed for reconstructing the VdM section age model. Panel A is plotted versus stratigraphic depth. Panels B-E are plotted versus age.

On the other hand, transfer of the time scale by Wang and collaborators [9] proved very straightforward. As each version of the age model was developed, the age of every sample was estimated by linear interpolation between the control points. We closely monitored changes in sedimentation rate when defining age-depth correlations. If substantial changes in sedimentation rates were generated by the use of specific age controls, we evaluated whether the implied changes in the flux of biogenic and/or detrital sediment were reasonable and justified within the geological setting of the VdM section. According to our age model, the studied record spans the time interval from ca. 870 ka to 740 ka (Table 2A and Fig. 3). For more information on *U. peregrina* oxygen isotope data, we refer to [3].

**Table 2:** A) Sample information and ostracod DCA sample axis 1 score obtained from the 51 species/34 samples matrix of Valle di Manche section (DCA performed with PAST 3.11). B) Bathymetric calibration of ostracod samples. Reduced major axis regression coefficients: slope a=0.46884; intercept b=24.175; r= -0.92; p=7.87 10^-6^; standard error of the estimates=14.3 m. C) Pearson linear correlation coefficient (r) and p (uncorr.) values (α=0.05) between DCA 1 sample scores and % of sand in each sample are shown. Regression models performed with PAST 3.11.

	**A) Ostracod Samples: age, grain size and DCA score**							**B) Water depth**
Label	Position (m)	Age (ka)	Sample weight (gr)	Sand fraction (>63 μm)		DCA1 sample score		Water depth (m)
				(gr)	(%)			
SMA53	12.81	741.8	46.9	2.67	5.7	164		101
SMA50	12.06	744.4	48.0	6.14	12.8	15		31
SMA46	11.06	747.8	50.8	1.44	2.8	4		26
SMA42	10.06	751.2	46.9	8.57	18.3	94		68
SMA38	9.06	754.6	48.8	3.26	6.7	135		87
SMA34	8.06	758.0	47.4	9.01	19.0	34		40
SMA30	7.06	761.2	45.4	14.39	31.7	79		61
SMA26	6.21	764.0	47.7	5.13	10.8	55		50
SMA22	5.31	767.0	54.9	1.48	2.7	41		43
SMA18	4.31	770.1	55.0	3.33	6.1	83		63
SMA14	3.31	773.4	57.2	3.20	5.6	242		138
SMA10	2.31	777.5	55.3	3.43	6.2	255		144
SMA8	1.81	780.0	56.2	5.11	9.1	235		134
SMA4	0.81	784.5	55.0	1.89	3.4	174		106
SMA2	0.31	786.3	58.5	1.52	2.6	238		136
SMA-1	0.00	787.5	51.8	2.58	5.0	151		95
SMA-8	−1.75	794.0	46.6	3.91	8.4	118		79
SMA-14	−3.25	795.6	46.4	6.79	14.6	80		62
SMB4	−4.25	796.7	54.9	11.41	20.8	0		24
SMB8	−5.25	797.7	57.1	7.82	13.7	2		25
SMB14	−6.75	799.3	56.2	3.52	6.3	20		34
SMB20	−8.25	800.9	55.1	4.81	8.7	38		42
SMB40	−14.00	811.6	54.0	4.96	9.2	45		45
SMB44	−15.00	813.8	50.1	19.76	39.4	20		34
SMB48	−16.00	827.3	54.5	21.30	39.1	66		55
SMB52	−17.00	839.4	55.5	6.05	10.9	91		67
SMB56	−18.00	846.3	55.0	18.22	33.1	134		87
SMB58	−18.50	850.6	53.8	6.11	11.4	240		137
SMB60	−19.00	855.8	56.4	6.79	12.0	267		149
SMB64	−20.00	861.9	53.5	1.30	2.4	204		120
SMB68	−21.00	863.6	54.9	6.80	12.4	162		100
SMB72	−22.00	865.3	54.9	4.57	8.3	52		49
SMB74	−22.50	866.1	50.4	4.05	8.0	25		36
SMB76	−23.00	867.0	54.6	8.54	15.6	24		35
	**C) DCA score*****vs*****. % of sand - linear correlation**							
			r = 0.291 r^2^ = 0.085 p = 0.094					

### 2.4 Environmental proxies calibration

Sand percentages within samples (a proxy for substrate texture) is interpreted as a driver of ostracod turnover along sedimentary successions. In this work, sand percentage was plotted against DCA axis-1 sample scores (Table 2A) via linear correlation (least squares) to evaluate the role of substrate in driving ostracod faunal changes (Table 2C). Sand fraction includes both biotic and abiotic grains >63 µm (Table 2A).

A linear correlation model (RMA) was also applied for bathymetry estimates of ostracod samples (Table 2B). Given the lack of quantitative water-depth information on ostracods species here recovered, water-depth calibrations rely on bathymetry inferences available for mollusc species retrieved in concomitance or proximity of the horizons sampled for ostracods (Table 1A column 3).

Sample-level bathymetry was calculated via the weighted average of a sub-set of extant mollusc species for which optimum bathymetry values were known (see Appendix 2 in [8]). Among the 14 extant taxa reported in [8], all cemented species (i.e., *Anomia ephippium, Heteranomia squamula*) were excluded from calibration, as they commonly show low association between ordination scores and bathymetry [13], [14]. Then, a RMA regression between sample-level bathymetry estimates and DCA axis-1 ostracod sample scores was calculated (Table 2B).

Information collected at Valle di Manche and relative climatic, environmental and chronostratigraphic inferences are summarised in Fig. 3.

## Funding sources

This research was funded by the University of Padova (Progetto di Ateneo 2011 and Dotazione Ordinaria della Ricerca (DOR) to LC) and University of Bologna (Ricerca Fondamentale Orientata, 2016 D. Scarponi).

## Appendix A Supplementary material

Appendix 1 – Ostracod Dataset with specimens counts for each sample. Symbol "*"= taxa and samples removed to generate 51 species and 34 sample matrix; "**" = removed to generate 40 species and 17 samples matrix.

## References

[1] V. Rossi, M. Azzarone, L. Capraro, C. Faranda, P. Ferretti, P. Macrì, D. Scarponi Response of benthic marine communities to Early-Middle Pleistocene environmental changes and sequence stratigraphic implications (Valle di Manche section, Southern Italy), Palaeogeogr. Palaeoclimatol. Palaeoecol. (in press).

[2] Capraro L., Macrì P., Scarponi D., Rio D. (2015). The lower to Middle Pleistocene Valle di Manche section (Calabria, Southern Italy): state of the art and current advances. Quat. Int..

[3] Capraro L., Ferretti P., Macrì P., Scarponi D., Tateo F., Fornaciari E., Bellini G., Dalan G. (2017). The Valle di Manche section (Calabria, Southern Italy): a high-resolution record of the Early-Middle Pleistocene transition (MIS 21-MIS 19) in the Central Mediterranean. Quat. Sci. Rev..

[4] Scarponi D., Azzarone M., Kowalewski M., Huntley J.W. (2017). Surges in trematode prevalence linked to centennial-scale flooding events in the Adriatic. Sci. Rep..

[5] Zuschin M., Nawrot R., Harzhauser M., Mandic O., Tomašových A. (2017). Taxonomic and numerical sufficiency in depth-and salinity-controlled marine paleocommunities. Paleobiology.

[6] R. Core Team, R: A language and environment for statistical computin*g. R Foundation for Statistical* Computing, Vienna, AustriaURL 〈https://www.R-project.org/〉, 2016.

[7] Hammer Ø., Harper D.A.T. (2005). Paleontological Data Analysis.

[8] Scarponi D., Huntley J.W., Capraro L., Raffi S. (2014). Stratigraphic paleoecology of the Valle di Manche section (Crotone Basin, Italy): a candidate GSSP of the Middle Pleistocene. Palaeogeogr. Palaeoclimatol. Palaeoecol..

[9] Wang P., Tian J., Lourens L.J. (2010). Obscuring of long eccentricity cyclicity in Pleistocene oceanic carbon isotope records. Earth Planet. Sci. Lett..

[10] Lourens L.J. (2004). Revised tuning of Ocean Drilling Program Site 964 and KC01B (Mediterranean) and implications for the d18O, tephra, calcareous nannofossil, and geomagnetic reversal chronologies of the past 1.1 Myr. Paleoceanography.

[11] Konijnendijk T.Y.M., Ziegler M., Lourens L.J. (2015). On the timing and forcing mechanisms of late Pleistocene glacial terminations: insights from a new high-resolution benthic stable oxygen isotope record of the eastern Mediterranean. Quat. Sci. Rev..

[12] Konijnendijk T.Y.M., Ziegler M., Lourens L.J. (2014). Chronological constraints on Pleistocene sapropel depositions from high-resolution geochemical records of ODP Sites 967 and 968. Newslett. Stratigr..

[13] Scarponi D., Kowalewski M. (2004). Stratigraphic paleoecology: bathymetric signatures and sequence overprint of mollusk associations from upper Quaternary sequences of the Po Plain, Italy. Geology.

[14] Wittmer J.M., Dexter T.A., Scarponi D., Amorosi A., Kowalewski M. (2014). Quantitative bathymetric models for late quaternary transgressive-regressive cycles of the Po Plain, Italy. J. Geol..

