# Supplementary material for: Early-Middle Pleistocene benthic turnover and oxygen isotope stratigraphy from the Central Mediterranean (Valle di Manche, Crotone Basin, Italy): Data and trends
Source: Data Brief. 2018 Feb 15;17:1099–107. doi: 10.1016/j.dib.2018.02.017 (PMC5988410; doi:10.1016/j.dib.2018.02.017)

05th February 2018

Dr. Michele Azzarone
Dipartimento di Scienze Biologiche, Geologiche e Ambientali,
Bologna University
Porta di Piazza San Donato 1
40126 Bologna
Italy
Email: michele.azzarone2@unibo.it

Conflict of Interest Statement

To the Managing Editor of Data in Brief

We reckon this study as particularly suited for Data in Brief, as it reports supporting information of a companion paper: Rossi et al., in press Paleo3 (PALAEO_8607). Data presented in this manuscript are original and have not been published previously.

Best regards,

Michele Azzarone

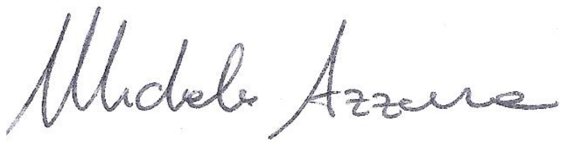

Supplement: Supplementary file 1 — Supplementary material [file mmc1.pdf]
